# Application of Potassium Improves Yield and Quality Under Drought Stress by Regulating Nutrient Use Efficiency in Wheat

**DOI:** 10.3390/plants15040539

**Published:** 2026-02-09

**Authors:** Jin Liu, Shuai-Bo Chen, Meng-Chuan Zhang, Yue Xiao, Bin Wang, Hai-Tao Liu, Peng-Fei Wang, Tian-Cai Guo, Guo-Zhang Kang, Ge-Zi Li

**Affiliations:** 1 The State Key Laboratory of High-Efficiency Production of Wheat-Maize Double Cropping, Henan Agricultural University, Zhengzhou 450046, China; liujin@henau.edu.cn (J.L.); chenshuaiboo@163.com (S.-B.C.); xiaoyue@stu.henau.edu.cn (Y.X.); wangbin@stu.henau.edu.cn (B.W.); wangpf@henau.edu.cn (P.-F.W.); guotiancai@henau.edu.cn (T.-C.G.); guozhangkang@henau.edu.cn (G.-Z.K.); 2 The National Engineering Research Center for Wheat, Henan Agricultural University, Zhengzhou 450046, China; zhangmengchuan@stu.henau.edu.cn; 3 College of Resources and Environment, Henan Agricultural University, Zhengzhou 450002, China; liuhaitaoky@henau.edu.cn; 4 The Henan Engineering Research Center of Functional Crop, Henan Agricultural University, Zhengzhou 450046, China

**Keywords:** *Triticum aestivum*, potassium, drought stress, yield, grain quality, nutrient use efficiency

## Abstract

Drought stress is a major abiotic constraint that severely limits growth, yield formation, and grain quality in wheat. Potassium (K) application is known to alleviate drought stress in crops. However, the integrated effects of K fertilization on yield, grain nutritional and quality, and nutrient use efficiency under post-flowering drought stress remains poorly understood. In this study, two-year pot experiments were conducted with two wheat cultivars under well-watered (75% field capacity) or post-flowering drought (~40% field capacity) conditions, combined with K fertilization (120 kg K_2_O ha^−1^) or no K (0 kg K_2_O ha^−1^), using a randomized complete block design. The results showed that K application significantly improved wheat performance under post-flowering drought stress. Specifically, it increased plant biomass, thousand-grain weight, grain yield, K accumulation, and K uptake efficiency, while also elevating grain contents of calcium, magnesium, zinc, total starch, and wet gluten. In conclusion, K fertilization not only mitigates the adverse effects of post-flowering drought stress in wheat, but also improves yield, grain quality, and nutrient use efficiency. These findings offer valuable insights for high-efficiency nutrient management and wheat production under drought regions.

## 1. Introduction

Wheat (*Triticum aestivum* L.) is one of the world’s most important cereal crops and a primary source of dietary carbohydrates and plant-based protein for humans [1,2]. In China, wheat ranks as the second most important food crop after rice, and its stable and high-yielding production is directly linked to national food security and socioeconomic stability [1]. However, the intensification of global climate change and the increasing frequency of extreme weather events have made drought a major abiotic stressor limiting wheat yield and stability [1,2]. In China, key wheat-producing regions—including the northern winter wheat zone and the northwestern and northeastern spring wheat zones—are chronically affected by seasonal drought [3]. Particularly during the critical reproductive stages from jointing to grain filling, water shortage severely impairs photosynthate translocation to grains, disrupts grain filling, reduces thousand-grain weight, and ultimately leads to yield losses ranging from 10% to 50% [4,5,6]. Therefore, the development of efficient, sustainable, and environmentally friendly strategies for drought mitigation and yield enhancement has become a central priority in agricultural research and production practices.

Currently, plants cope with water-deficit stress through mechanisms such as osmotic adjustment, stomatal regulation, antioxidant defense, and root system remodeling. Beyond genetic improvement, the application of exogenous substances is also an effective strategy; small molecules such as plant hormones (e.g., abscisic acid, jasmonates, etc.), nutrients, amino acids, and osmolytes can act as signaling molecules or metabolic primers to enhance stress resistance [7]. Among various strategies, nutrient management has attracted considerable attention due to its operational simplicity, low cost, and environmental compatibility [8]. Potassium (K), as one of the essential macronutrients for plants, plays an irreplaceable role in growth, development, and stress responses [9,10]. Although K is not a structural component of organic molecules, it participates critically in key physiological processes, including osmotic regulation, enzyme activation, charge balance, photoassimilate transport, and signal transduction [11]. Under water-deficit conditions, K enhances crop drought tolerance through multiple mechanisms, including the maintenance of cell turgor, regulation of stomatal aperture, strengthening of antioxidant defense systems, and promotion of root development [12]. For instance, in sesame, K mitigates oxidative damage and sustains carbon assimilation by modulating the activity and gene expression of sucrose metabolism-related enzymes [13]; in melon, it improves leaf water potential, chlorophyll stability, and antioxidant capacity, thereby maintaining photosynthetic efficiency under drought [14]; in sorghum, K alleviates water stress by enhancing root hydraulic conductivity and leaf relative water content [15]; and in maize, exogenous K up-regulates catalase (CAT) activity and promotes the accumulation of osmoprotectants such as proline, soluble sugars, and proteins, which helps stabilize cellular structures and maintain metabolic homeostasis [16]. Moreover, K fertilization has been consistently shown to improve both yield and grain quality across multiple crops. It increases panicle grain number and grain plumpness in rice [17], enhances hundred-grain weight and starch content in maize [18], and improves dry matter and β-carotene levels in sweet potato storage roots [19]. Additionally, the viscoelastic network formed by gluten proteins is critical for determining the rheological properties of dough and the processing quality of fermented foods like bread. Notably, studies have indicated that application of K can enhance key quality indicators in wheat, such as crude protein content [20].

Despite its well-established physiological roles, K fertilization has long been undervalued in practical nutrient management compared to nitrogen (N) and phosphorus (P) [9]. This issue stems largely from a widespread misconception that most agricultural soils are naturally rich in available K, especially in areas underlain by K-bearing minerals such as feldspar or mica. However, this view overlooks both the dynamic nature of soil K pools and the generally low bioavailability of most soil K [9,21]. The slow release of non-exchangeable K into the exchangeable pool often fails to meet the high demand of modern high-yielding wheat varieties, especially in intensive cropping systems with high cropping intensity and short or absent fallow periods [1,9]. This “N- and P-heavy, K-light” fertilization pattern not only limits wheat yield potential but also weakens the crop’s ability to cope with abiotic stresses like drought; therefore, science-based K management is critical for enhancing soil fertility and developing climate-resilient agricultural systems. Nevertheless, most existing studies have focused on K fertilization effects across the entire growing season or during vegetative stages. However, attention is rarely paid to the roles between application of K and the grain yield, nutrient use efficiency, and grain quality under post-flowering drought stress in wheat. Given that the reproductive phase in wheat, particularly from anthesis to grain filling, represents its most drought-sensitive window, which is characterized by high transpirational demand and active photoassimilate partitioning to grains; thus, it is imperative to clarify the role of K fertilization during this stage. Therefore, this pot experiment specifically investigates post-flowering drought stress and aims to systematically address the following three core scientific questions: (1) How does application of K fertilization influence grain yield and its components in wheat? (2) How does application of K fertilization regulate the uptake and utilization efficiency of K and other essential mineral nutrients (e.g., Ca, Mg, Zn)? (3) What are the effects of K fertilization on key grain quality parameters, such as starch and wet gluten content?

The findings of this pot experiment will provide a theoretical understanding of how K and water interact to regulate wheat physiology and ecology in the reproductive stage, Furthermore, they will offer scientific support for formulating green and efficient K-based fertilization strategies aimed at enhancing drought resilience, stabilize yield, and improve quality in arid and semi-arid wheat growing regions.

## 2. Results

### 2.1. Application of K Fertilization Improves Wheat Growth and Yield

**Figure 1:**
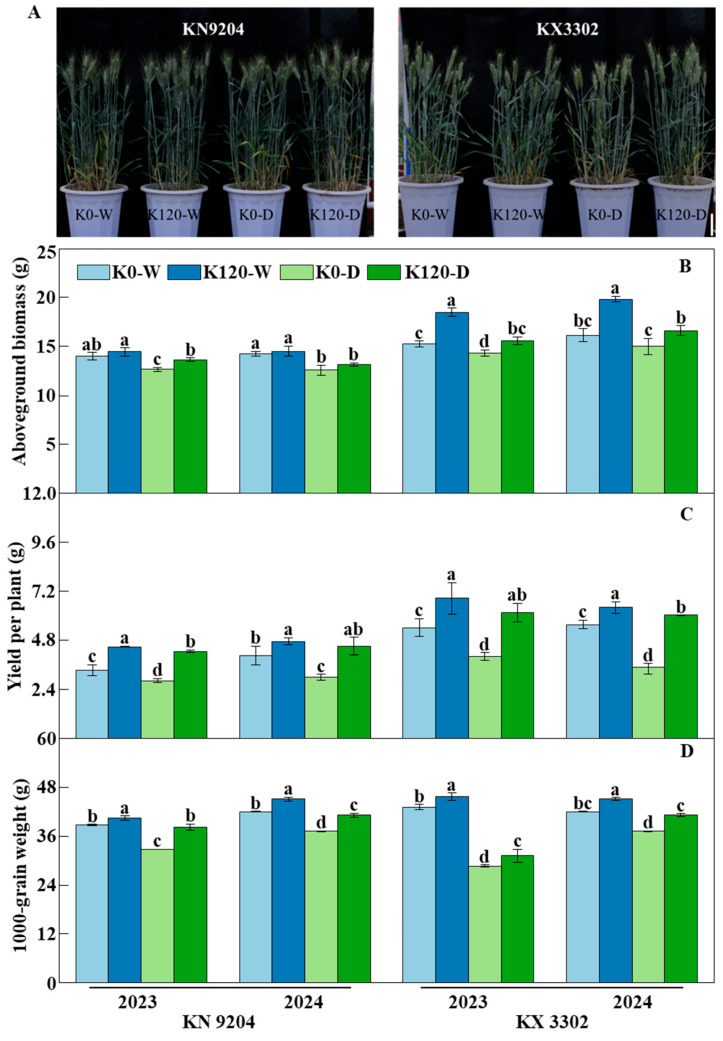
Phenotypic responses and agronomic performance of wheat cultivars KX3302 and KN9204 under potassium and drought treatments. (**A**) Representative phenotypes of KX3302 and KN9204 under well-watered (W) and post-flowering drought stress (D) conditions, with or without potassium fertilization (K120 or K0). Bar = 10 cm. (**B**) Aboveground biomass per plant of KX3302 and KN9204 in 2023 and 2024 under four treatment combinations: K0-W, K120-W, K0-D, and K120-D. (**C**) Grain yield per plant of both cultivars across the same four treatments in 2023 and 2024. (**D**) Thousand-grain weight (TGW) of KX3302 and KN9204 under the four treatments in both years. K0-W: well-water condition and not K fertilization treatment; K120-W: well-water condition and K fertilization treatment; K0-D: post-flowering water-limited conditions and not K fertilization treatment; K120-D: post-flowering water-limited conditions and K fertilization treatment; Data present means ± SE (*n* = 3). Different lowercase letters indicate significant differences (*p* < 0.05) among treatments within the same year and cultivar.

To investigate the effects of K fertilization on wheat, a two-year pot experiment (2022–2024) was conducted with two K fertilization treatments (K0: 0 kg K_2_O ha^−1^; K120: 120 kg K_2_O ha^−1^) and two cultivars (KN9204 and KX3302). Results showed that K fertilization significantly increased aboveground biomass throughout the growing season (Figure 1B), whereas plant height remained unaffected (Figure 1A). The analysis of variance showed that year, cultivar, treatment, and their interactions had significant effects on the vast majority of the measured indices (Supplementary Table S1). Therefore, to accurately interpret the treatment effects, all results in this study are presented separately by year and cultivar. Under well-watered conditions, K120 increased biomass to 1.34 and 1.17 times in KN9204, and to 1.15 and 1.17 times in KX3302 in 2023 and 2024, respectively, compared to K0. Under post-flowering drought stress, K fertilization also markedly enhanced biomass; increases to 1.50 and 1.51 times were observed in KN9204, and 1.14 and 1.09 times in KX3302 in 2023 and 2024, respectively. Furthermore, under K120, drought stress reduced biomass to 0.94 and 0.91 times in KN9204, and to 0.88 and 0.85 times in KX3302 in 2023 and 2024, respectively. In contrast, under K0, drought-induced biomass reductions were 0.90 and 0.88 times for KN9204, and 0.90 and 0.91 times for KX3302 (Figure 1B).

Regarding grain yield, the results showed that K fertilization significantly increased grain yield per plant and 1000-grain weight (TGW) under the two K fertilization treatments, while no significant differences were observed in spike number per plant or grain number per spike (Figure S1 and Figure 1). Under well-watered conditions, compared with the K0, the K120 treatment increased grain yield per plant to 1.34 and 1.17 times in KN9204, and to 1.26 and 1.15 times in KX3302, in 2023 and 2024, respectively (Figure 1C). TGW also increased to 1.05 and 1.07 times in KN9204, and to 1.06 and 1.07 times in KX3302. Under post-flowering drought, K fertilization still significantly improved yield; grain yield rose to 1.50 times (2023) and 1.51 times (2024) in KN9204, and to 1.54 and 2.29 times in KX3302; TGW increased to 1.17 and 1.11 times in KN9204, and to 1.09 and 1.11 times in KX3302 (Figure 1D). In the K120 treatment, drought stress decreased grain yield to 0.95 times (2023) and 0.95 times (2024) in KN9204, with TGW reductions to 0.94 and 0.91 times; in KX3302, yield declined to 0.90 and 0.94 times, and TGW to 0.68 and 0.91 times. Under K0, drought caused more severe yield losses; grain yield dropped to 0.85 and 0.74 times in KN9204, and to 0.74 and 0.47 times in KX3302; TGW decreased to 0.85 and 0.89 times in KN9204, and to 0.67 and 0.89 times in KX3302, respectively (Figure 1C,D). Taken together, despite variations between different years and cultivars, potassium application exhibited a stable and consistent positive trend in enhancing wheat biomass, grain yield per plant, and thousand-grain weight. Specifically, analysis of variance indicated that grain yield per plant and TGW were significantly regulated by cultivar, year, treatment, and their interactions, whereas aboveground biomass responded primarily to the treatment (Supplementary Table S1).

### 2.2. Application of K Fertilization Improves the Content, Accumulation, Absorption, Harvest Index, and Utilization Efficiency of K in Wheat

**Figure 2:**
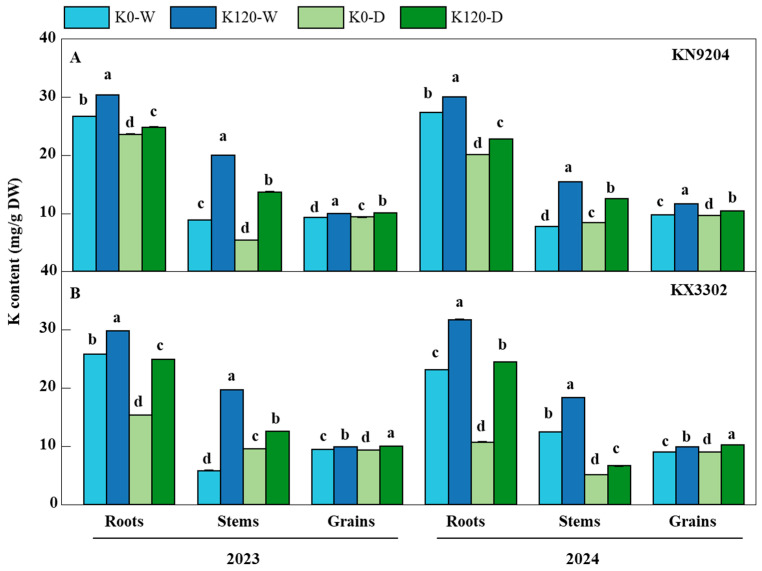
K content in roots, stems, and grains of wheat cultivars KN9204 and KX3302 under four treatment combinations across 2023 and 2024. (**A**) K content in roots, stems, and grains of KN9204 under K0-W (well-watered, no K fertilization), K120-W (well-watered and K fertilization treatment), K0-D (post-flowering drought, no K fertilization), and K120-D (post-flowering drought and K fertilization treatment) treatments in 2023 and 2024. (**B**) K content in roots, stems, and grains of KX3302 under the same four treatments in both years. Data are presented as means ± SE (*n* = 3). Different lowercase letters above bars indicate significant differences among treatments within the same tissue and year at *p* < 0.05.

To investigate whether K fertilization influences K accumulation, uptake, harvest index, and utilization efficiency in different wheat tissues, K content and related indices were measured in this pot experiment (Figure 2, Table 1). Results showed that K fertilization significantly altered K distribution and use dynamics in wheat plants. Under well-watered conditions, compared to the K0 treatment, K120 increased K content in stems to 2.25 and 1.99 times, in roots to 1.14 and 1.10 times, and in grains to 1.07 and 1.21 times in KN9204 in 2023 and 2024, respectively. Aboveground K accumulation (AKA) rose to 1.94 and 1.75 times, grain K accumulation (GKA) to 1.43 and 1.28 times, and K uptake efficiency (KUPE) to 1.94 and 1.75 times. However, K harvest index (KHI) decreased to 0.74 and 0.81 times, aboveground K use efficiency (AKUE) to 0.53 and 0.58 times, and grain K use efficiency (GKUE) to 0.69 and 0.67 times. In KX3302, K120 led to modest increases in grain K content (1.05 and 1.09 times) but markedly enhanced GKA (1.32 and 1.16 times), AKA (2.75 and 1.70 times), and KUPE (2.75 and 1.07 times). Notably, KHI decreased to 0.48 and 0.74 times, GKUE to 0.46 and 0.68 times, and AKUE to 0.44 and 0.72 times (Table 1).

Under post-flowering drought stress, K120 still elevated K content in KN9204 stems (2.54 and 1.50 times), roots (1.05 and 1.13 times), and grains (1.07 times), while dramatically increasing AKA (2.15 and 1.42 times), GKA (1.61 and 1.80 times), and KUPE (2.15 and 1.42 times). However, KHI decreased to 0.75 times in 2023, and both GKUE (0.70 times) and AKUE (0.50 and 0.73 times) were reduced. In KX3302 under drought, K fertilization increased grain K content to 1.07 (2023) and 1.14 (2024), and substantially improved GKA (1.65 and 2.42 times), AKA (1.32 and 1.51 times), KUPE (1.32 and 1.51 times), KHI (1.25 and 1.73 times), and GKUE (1.16 and 1.52 times), although AKUE decreased to 0.82 and 0.73 times (Table 1). The results indicated that the effects of potassium application on increasing potassium content and potassium-related efficiency indices in wheat were consistent across different years and cultivars. Further in-depth analysis of variance revealed that these indices were significantly influenced by treatment, year × cultivar, year × treatment, cultivar × treatment, and year × cultivar × treatment interactions (Supplementary Table S1).

### 2.3. Application of K Fertilization Changes Mineral Content and Grain Quality in Wheat

To further investigate the effects of K fertilization on grain quality, mineral content—including calcium (Ca), magnesium (Mg), manganese (Mn), iron (Fe), and zinc (Zn)—were measured in mature grains (Table 2). Results showed that K fertilization differentially modulated mineral accumulation between cultivars: in KX3302, K120 significantly increased Ca (+1.06 times in 2023, +1.04 times in 2024), Mg (+1.12 times and +1.04 times), and Mn (only in 2023); whereas in KN9204, K fertilization reduced Ca and Mg content but enhanced Fe (in 2023) and Zn (in both years). These findings indicate that K fertilization can reshape grain mineral composition. Subsequent analysis of key quality traits revealed that K120 significantly improved total starch, protein, and wet gluten contents (Table 2). Under well-watered conditions, total starch increased to 1.03 and 1.07 times in KN9204, and to 1.07 and 1.23 times in KX3302 in 2023 and 2024, respectively. Protein content rose to 1.07 and 1.13 times in KN9204, and to 1.05 and 1.03 times in KX3302; wet gluten content increased to 1.07 and 1.11 times in KN9204, and to 1.05 and 1.04 times in KX3302 (Table 2).

Under post-flowering drought stress, K fertilization still markedly enhanced quality traits in KN9204; total starch increased to 1.09 (2023) and 1.27 times (2024), protein to 1.09 and 1.09 times, and wet gluten to 1.10 and 1.08 times (Table 3). In contrast, improvements in KX3302 were modest—total starch, protein, and wet gluten rose to only 1.06 and 1.03 times, 1.02 and 1.03 times, and 1.01 and 1.02 times, respectively (Table 3). Notably, under drought stress, total starch in the K120 treatment declined to 0.97 times and 1.00 times in KN9204, and to 0.99 and 0.96 times in KX3302 compared to well-watered K120. However, protein and wet gluten contents increased under drought; in KN9204, protein rose to 1.07 and 1.02 times, and wet gluten to 1.06 and 1.03 times; in KX3302, protein increased to 1.01 and 1.02 times, and wet gluten to 1.00 and 1.03 times (Table 3). The above results indicate that although different treatment exhibited variations for different mineral elements across years and cultivars, an overall stable and consistent trend was observed. Specifically, analysis of variance showed that the contents of each element (Ca, Mg, Mn, Fe, and Zn) were significantly influenced by year, cultivar, treatment, and their complex interactions (Supplementary Table S1).

### 2.4. Correlation Analysis Between Various Indexes and K Utilization Efficiency in Wheat

**Figure 3:**
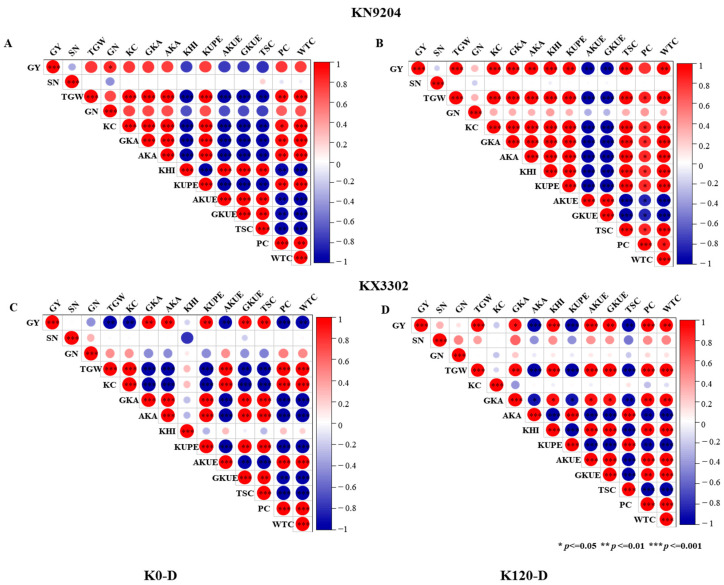
Correlation analysis between grain yield and multiple agronomic and quality traits in wheat cultivars KN9204 and KX3302. Panels (**A**,**B**) show results for KN9204 under K0–D, and K120–D, respectively; panels (**C**,**D**) present corresponding data for KX3302 under the same treatments. Significant correlations (*p* < 0.05) are color-coded (red for positive, blue for negative), with circle size proportional to the absolute value of the correlation coefficient (r).

To elucidate the effects of K fertilization on the relationships between grain yield per plant and multiple agronomic and quality traits, correlation analyses were conducted for the two wheat cultivars KN9204 and KX3302 (Figure 3). The results revealed distinct correlation patterns between grain yield per plant (GY) and other traits under different water and K regimes: under well-watered conditions without K fertilization (K0), GY in KN9204 and KX3302 showed no highly significant correlations (*p* ≤ 0.01) with any of the 13 measured traits, whereas in KX3302, it was significantly positively correlated only with grain K use efficiency (GKUE) (*p* ≤ 0.01) (Figure S2); under well-watered K120 treatment, GY in KN9204 was highly positively correlated with thousand-grain weight (TGW), stem K content (KC), grain K accumulation (GKA), K harvest index (KHI), GKUE, total starch content (TSC), protein content (PC), and wet gluten content (WTC) (*p* ≤ 0.01), while in KX3302, yield per plant was significantly positively correlated with TGW, GKA, KHI, aboveground K use efficiency (AKUE), GKUE, and TSC (*p* ≤ 0.01) (Figure S2); under post-flowering drought stress without K fertilization (K0), KN9204 and KX3302 still exhibited no highly significant correlations (*p* ≤ 0.01) (Figure 3); under drought stress with K120 fertilization, GY in KN9204 was highly positively correlated with TGW, KC, GKA, aboveground K accumulation (AKA), KHI, K uptake efficiency (KUPE), TSC, and WTC (*p* ≤ 0.01), while in KX3302, it was significantly positively correlated with TGW, KHI, AKUE, GKUE, PC, and WTC (*p* ≤ 0.01). Collectively, K fertilization enhances yield stability and grain quality under drought stress by modulating K uptake and utilization efficiency (Figure 3).

## 3. Discussion

### 3.1. K Fertilization Mitigates Drought-Induced Yield Loss by Enhancing Biomass Accumulation and Grain Filling

Potassium is a pivotal macronutrient that plays a central role in osmoregulation, stomatal regulation, and enzyme activation—processes critical for plant resilience under abiotic stress [22]. Consistent with previous findings [23,24], our pot experiment demonstrates that K fertilization (K120) significantly enhances plant biomass, spike number, 1000-grain weight (TGW), and grain yield in wheat under both well-watered and post-flowering drought conditions. Notably, while drought stress invariably reduced these traits—as widely reported [25,26,27]—the magnitude of reduction was greater in the K0 treatment than in K120. This indicates that K fertilization buffers the adverse effects of water deficit during the grain-filling stage, likely by maintaining cell turgor, improving photosynthetic efficiency, and sustaining carbohydrate translocation to developing grains [28]. Beyond affecting yield-related traits, potassium directly regulates grain quality. As a cofactor for key enzymes in protein synthesis, potassium may promote gluten protein aggregation by influencing the ionic environment, thereby enhancing gluten network strength and elasticity [29]. Our study found that potassium application significantly increased wet gluten content (Table 3). These results confirm that potassium improves wheat quality and highlight its key role in mitigating post-flowering drought stress. It is noteworthy that neither potassium fertilization nor post-anthesis drought stress significantly altered plant height at maturity (Figure 1A). This phenomenon can be attributed to the timing of the imposed stress. Plant height is primarily determined during the vegetative and stem-elongation stages, which occurred prior to our drought treatment initiated after anthesis. Consequently, the post-anthesis drought primarily affected grain-filling and assimilate partitioning processes rather than vegetative growth. These results demonstrate that the impacts of potassium and drought on biomass, yield components, and grain quality (Figure 1B–D and Figure 2; Table 1, Table 2 and Table 3) were predominantly driven by physiological adjustments in assimilation, partitioning, and stress tolerance during the reproductive phase, rather than by changes in plant architecture.

### 3.2. K Fertilization Optimizes K Use Efficiency and Modulates Mineral Nutrient Distribution in Grains

Beyond its direct agronomic benefits, K fertilization profoundly influences nutrient use efficiency and grain mineral composition. Our results are consistent with Raza et al. (2013) and Matthew et al. (2013) [30,31], demonstrating that K120 significantly increased aboveground K accumulation (AKA), grain K accumulation (GKA), K uptake efficiency (KUPE), and K harvest index (KHI) under well-watered conditions, and maintains high GKA and KUPE even under drought stress (Table 1). These improvements reflect enhanced root uptake capacity and efficient internal K remobilization from vegetative tissues to grains—a key determinant of yield stability under stress [32]. Intriguingly, K fertilization also altered the accumulation of other essential minerals; in KX3302, grain Ca and Mg contents increased under both well-watered and drought conditions, while Fe and Zn were elevated only under well-watered K120 (Table 2). Such interactions underscore the importance of balanced K nutrition not only for yield but also for biofortification strategies aimed at improving grain nutritional quality.

### 3.3. K Fertilization Affects Grain Quality Traits by Under Drought Stress

Grain quality in wheat—particularly starch and protein profiles—is highly sensitive to environmental fluctuations, with drought typically suppressing starch synthesis while elevating protein concentration due to reduced carbohydrate availability and concentrated nitrogen [33]. In this context, our finding that K120 significantly boosts total starch, protein, and wet gluten content under both water regimes introduces a crucial nuance: K fertilization not only mitigates yield loss but actively improves end-use quality. Remarkably, the positive effect on starch accumulation under drought was more pronounced in KX3302 than in KN9204, suggesting a potential cultivar-specific synergy between K nutrition and carbon partitioning under stress. This difference may be related to the potassium uptake capacity between KX3302 and KN9204. Potassium is a key regulator in stress adaptation, primarily through its roles in osmoregulation, enzyme activation, and charge balance—processes that directly influence photosynthesis, assimilate partitioning, and cellular homeostasis under water deficit [10]. The stronger starch response in KX3302 could therefore reflect a genotype-specific capacity to leverage K-mediated osmoregulation or to maintain higher activity of K-dependent enzymes involved in starch biosynthesis under stress. These mechanisms, while not directly measured here, offer plausible physiological explanations for the observed genotypic responses to K under drought. Meanwhile, the consistent increase in wet gluten across both cultivars aligns with K’s role in stabilizing protein structure and dough rheology. Collectively, these results suggest that K fertilization may serve as a dual-purpose strategy—simultaneously enhancing yield resilience and functional grain quality—holds particular promise for drought-prone regions where both productivity and nutritional value are at risk.

## 4. Materials and Methods

### 4.1. Experimental Material and Experimental Design

A pot experiment was conducted during the wheat-growing seasons from 2022 to 2024 under a rainout shelter at the Science and Education Demonstration Park of Henan Agricultural University, Zhengzhou (34°51′ N, 113°35′ E), using two winter wheat cultivars-Kenong 9204 (KN9204) and Kexing 3302 (KX3302). These two wheat varieties are high-yield varieties with wide cultivation adaptability and yield stability in the current Huang-Huai winter wheat area. This pot experiment uniformly used locally typical sandy loam soil. The K0 treatment utilized soil from the experimental field that had not received potassium fertilizer for an extended period, while the K120 treatment used soil with normal potassium application. The soil, collected from the 0–20 cm plow layer, contained 117.8 mg kg^−1^ alkaline hydrolyzable nitrogen, 23.7 mg kg^−1^ available phosphorus, 181.7 mg kg^−1^ available potassium, and 13.2 g kg^−1^ organic matter. Each pot was filled with 10 kg of air-dried soil. At sowing, a basal fertilization was uniformly applied to all pots; phosphorus was supplied as triple superphosphate (Ca(H_2_PO_4_)_2_) at a rate of 1.35 g P_2_O_5_ per pot, and nitrogen was supplied as urea (CO(NH_2_)_2_) at a rate of 1.15 g N per pot. For the potassium fertilization treatments, the K120 group additionally received potassium sulfate (K_2_SO_4_) as a basal dose at a rate of 1.15 g K_2_O per pot, while the no-potassium control group (K0) received no potassium fertilizer. The fertilization setup referred to [34]. A topdressing of 1.15 g nitrogen was applied to all pots at the stem elongation stage. Seedlings were thinned to 12 uniform plants per pot at the three-leaf stage. All pots were placed within the same rainout shelter throughout the growth period to completely exclude natural precipitation. Drought stress was initiated at anthesis and maintained until maturity using a gravimetric method to control two water regimes: well-watered (W, 75% field capacity) and drought stress (D, 45% field capacity) [35,36]. To achieve precise water control, all pots were weighed individually every day. Water was replenished based on daily weight loss and the target soil moisture, calculated and applied uniformly by treatment group (well-watered or drought-stressed) to maintain the set soil moisture levels. During the experimental period, the monthly average temperature ranged from 21 °C to 33 °C in 2023, with an average relative humidity of 60%; whereas in 2024, the monthly average temperature ranged from 23 °C to 36 °C, with an average relative humidity of 57%. The field management during wheat growth was consistent with the field test.

### 4.2. Agronomic Characters Measurement

Plant phenotypes were photographed periodically throughout the wheat growth cycle to document growth and stress responses. At physiological maturity (approximately 40–45 days after anthesis), five representative plants per treatment were randomly selected to measure plant height (from soil surface to the tip of the main spike, in cm), aboveground biomass per plant (oven-dried at 80 °C to constant weight, in g), grain yield per plant (after threshing and air-drying, in g), number of spikes per plant, and grain number per spike (manually counted). The mean of the five biological replicates was used for statistical analysis.

### 4.3. K and Other Elements Content Measurement

Aboveground samples were collected at physiological maturity, oven-dried at 80 °C to constant weight, and ground to pass through a 0.5 mm sieve. Samples were digested using a HNO_3_–H_2_O_2_ microwave-assisted digestion system. Content of K and other mineral elements—including calcium (Ca), magnesium (Mg), zinc (Zn), Iron (Fe), manganese and (Mn), and copper (Cu)—were determined by inductively coupled plasma mass spectrometry (ICP-MS 7900; Agilent, Santa Clara, USA), following the protocol described by Liu et al. (2025) [37]. Three biological replicates per treatment were analyzed, and results are expressed as mg·g^−1^ on a dry weight basis.

### 4.4. Calculation of K Efficiencies

K efficiency indices were calculated following Wang et al. (2017), Yang et al. (2021), and Sun et al. (2024) [28,32,38]: Aboveground K Accumulation (AKA, kg ha^−1^), Grain K Accumulation (GKA, kg ha^−1^), K Harvest Index (KHI, %), K Uptake Efficiency (KUPE, %), Aboveground K Use Efficiency (AKUE, kg kg^−1^), Grain K Use Efficiency (GKUE, kg kg^−1^), K Agronomic Efficiency (KAE, kg kg^−1^), and K Partial Factor Productivity (KPFP, kg kg^−1^). The formulas are as follows:

AKA = aboveground K content × dry weight of aboveground

GKA = grain K content × dry weight of grain

KHI = grain K accumulation/aboveground K accumulation

KUPE = above ground K accumulation at maturity/K fertilizer application

AKUE = above ground K accumulation in K120 − above ground K accumulation in K0/K fertilizer application

GKUE = grain K accumulation in K120 − grain K accumulation in K0/K fertilizer application

KAE = grain yield in K120 − grain yield in K0/K fertilizer application

KPFP = grain yield/K fertilizer application

### 4.5. Determination of Protein Content

The seed protein content in this experiment was determined using a Perten IM9500 (Perten Instruments, Hägersten, Sweden)multifunctional near-infrared grain analyzer [39]. Three biological replicates per treatment were analyzed, and mean values were used for statistical evaluation.

### 4.6. Determination of Starch Component Content

Total starch content was determined by polarimetry based on AOAC Method 996.11 (AOAC Official Method 996.11) with minor modifications. Briefly, 0.75 g of whole-wheat flour (with known moisture content) was hydrolyzed in 7.5 mL of 0.32 M HCl at boiling for 10 min and rapidly cooled. Proteins were precipitated by sequential addition of 0.9 mL of 3% (*w*/*v*) zinc sulfate and 0.9 mL of 15% (*w*/*v*) K ferrocyanide with vigorous mixing. The mixture was diluted to 10 mL, allowed to stand for 10 min, and centrifuged at 3000× *g* for 5 min. The optical rotation (α) of the supernatant was measured using a polarimeter (WZZ-2S, Shanghai Shenguang, China) with a 1 dm tube, and total starch (%) was calculated as:

Total starch (%) = [α × 15/(L × 203 × m × (1 − M))] × 100

where α = observed rotation (°), L = tube length (dm; 1 in this study), 203 = specific rotation of pure starch, m = sample mass (g), and M = moisture content (decimal). Three biological replicates per treatment were analyzed, and mean values were used for statistical evaluation.

### 4.7. Determination of Wet Gluten Content

Wet gluten content was determined according to AACC Method 38–12. Briefly, 10 g of whole-wheat flour (adjusted to 14% moisture basis) was mixed with 4.5 mL of 2% NaCl solution and hand-kneaded into a dough, which was then rested for 20 min. The dough was gently washed under running tap water at room temperature until the effluent was neutral and starch-free. The resulting wet gluten mass was dewatered by centrifugation (3000× *g*, 2 min) and immediately weighed. Results were expressed as a percentage of the original flour weight on a dry basis.

### 4.8. Data Analysis

The experiment followed a completely randomized design with a multi-factor (cultivar × treatment (the full combination of water regime and K fertilization) × year) factorial arrangement. Data were analyzed using three-way analysis of variance (ANOVA) to assess the main effects of cultivar, treatment and year, as well as their interactions. Where significant interactions were found (Supplementary Table S1), means among treatment combinations within the same cultivar and year were subsequently separated using Fisher’s Least Significant Difference (LSD) test. Data were analyzed using IBM SPSS Statistics 25 and presented as mean ± standard error (SE). Graphs were generated with Origin 2021.

## 5. Conclusions

Drought stress severely impairs plant growth, nutrient uptake and translocation, and ultimately grain yield and quality. K fertilization plays a pivotal role in mitigating these adverse effects. Our two-year pot experiments demonstrated that, under the specific pot-based system and soil conditions used in this study, K fertilization at K120 significantly enhanced biomass accumulation, grain yield, and potassium use efficiency—including K uptake, remobilization, and harvest index—under both well-watered and post-flowering drought conditions. Furthermore, K120 improved key grain quality, such as total starch, protein, and wet gluten content, especially under water-limited environments. These findings underscore that, under similar soil and management conditions, K fertilization represents an effective agronomic strategy for simultaneously bolstering resilience and grain quality in wheat under increasing drought risk under climate change.

## Figures and Tables

**Table 1:** Effect of K fertilization on content, accumulation, absorption, harvest index, and utilization efficiency of K in wheat.

Variety	Year	Treatment	KUPE (g/g)	GKA (g/plant)	AKA (g/plant)	KHI (%)	GKUE (g/g)	AKUE (g/g)
KN 9204	2023	K0-W	0.118 ± 0.001 c	31.149 ± 0.089 c	125.272 ± 1.002 c	24.867 ± 0.254 c	0.027 ± 0.000 b	0.112 ± 0.001 b
		K120-W	0.229 ± 0.002 a	44.677 ± 0.082 a	242.851 ± 1.795 a	18.398 ± 0.169 d	0.018 ± 0.000 d	0.060 ± 0.000 d
		K0-D	0.074 ± 0.001 d	26.488 ± 0.050 d	78.666 ± 1.174 d	33.680 ± 0.552 a	0.036 ± 0.001 a	0.161 ± 0.002 a
		K120-D	0.160 ± 0.003 b	42.642 ± 0.105 b	169.147 ± 2.854 b	25.218 ± 0.481 b	0.025 ± 0.000 c	0.081 ± 0.001 c
	2024	K0-W	0.112 ± 0.008 c	38.877 ± 0.079 c	118.311 ± 7.971 c	33.014 ± 2.290 a	0.034 ± 0.002 a	0.121 ± 0.008 a
		K120-W	0.195 ± 0.003 a	49.561 ± 0.081 b	206.603 ± 3.167 a	26.658 ± 0.400 c	0.023 ± 0.000 d	0.070 ± 0.001 d
		K0-D	0.103 ± 0.001 d	29.044 ± 0.006 d	109.202 ± 0.670 d	26.598 ± 0.161 d	0.027 ± 0.000 c	0.115 ± 0.001 b
		K120-D	0.147 ± 0.000 b	52.295 ± 0.027 a	155.608 ± 0.077 b	30.249 ± 0.034 b	0.029 ± 0.000 b	0.085 ± 0.000 c
KX 3302	2023	K0-W	0.102 ± 0.001 d	51.155 ± 0.027 c	108.174 ± 0.586 d	47.290 ± 0.266 a	0.050 ± 0.000 a	0.141 ± 0.001 a
		K120-W	0.281 ± 0.003 a	67.725 ± 0.094 a	297.486 ± 3.629 a	22.769 ± 0.298 d	0.023 ± 0.000 d	0.062 ± 0.001 d
		K0-D	0.128 ± 0.002 c	37.221 ± 0.065 d	136.039 ± 2.320 c	27.368 ± 0.419 c	0.029 ± 0.001 c	0.105 ± 0.002 b
		K120-D	0.170 ± 0.000 b	61.407 ± 0.072 b	179.805 ± 0.072 b	34.152 ± 0.026 b	0.034 ± 0.000 b	0.087 ± 0.000 c
	2024	K0-W	0.172 ± 0.000 b	54.568 ± 0.087 c	182.176 ± 0.151 b	27.649 ± 0.060 b	0.031 ± 0.000 b	0.089 ± 0.000 c
		K120-W	0.292 ± 0.000 a	63.176 ± 0.192 a	309.254 ± 0.250 a	20.428 ± 0.064 c	0.021 ± 0.000 c	0.064 ± 0.000 d
		K0-D	0.082 ± 0.003 d	25.516 ± 0.097 d	86.989 ± 3.620 d	27.269 ± 1.168 b	0.030 ± 0.001 b	0.173 ± 0.007 a
		K120-D	0.124 ± 0.001 c	61.844 ± 0.102 b	131.260 ± 1.116 c	47.119 ± 0.342 a	0.046 ± 0.000 a	0.126 ± 0.001 b

Note: Data present means ± SE (*n* = 3). Different lowercase letters indicate significant differences (*p* < 0.05) among treatments within the same year and cultivar. KUPE, K uptake efficiency; GKA, grain K accumulation; AKA, aboveground K accumulation; KHI, K harvest index; GKUE, grain K use efficiency; AKUE, aboveground K use efficiency.

**Table 2:** Effect of K fertilization on mineral content in wheat.

Variety	Year	Treatment	Ca (mg/g)	Mg (mg/g)	Fe (mg/g)	Mn (mg/g)	Zn (mg/g)
KX 3302	2023	K0-W	0.285 ± 0.031 b	0.605 ± 0.048 b	0.0320 ± 0.001 a	0.0109 ± 0.001 c	0.0145 ± 0.000 c
		K120-W	0.301 ± 0.036 a	0.679 ± 0.084 a	0.0317 ± 0.002 ab	0.0122 ± 0.000 b	0.0169 ± 0.001 b
		K0-D	0.280 ± 0.027 c	0.603 ± 0.029 b	0.0306 ± 0.002 c	0.0093 ± 0.001 d	0.0137 ± 0.001 d
		K120-D	0.279 ± 0.030 c	0.678 ± 0.077 a	0.0316 ± 0.001 b	0.0128 ± 0.001 a	0.0182 ± 0.001 a
	2024	K0-W	0.303 ± 0.024 b	0.674 ± 0.029 b	0.0364 ± 0.001 a	0.0128 ± 0.000 b	0.0172 ± 0.001 a
		K120-W	0.315 ± 0.042 a	0.702 ± 0.061 a	0.0362 ± 0.004 a	0.0108 ± 0.001 d	0.0159 ± 0.001 c
		K0-D	0.290 ± 0.012 c	0.676 ± 0.033 b	0.0350 ± 0.000 b	0.0135 ± 0.000 a	0.0171 ± 0.001 a
		K120-D	0.300 ± 0.024 b	0.707 ± 0.053 a	0.0334 ± 0.000 c	0.0126 ± 0.001 c	0.0163 ± 0.000 b
KN 9204	2023	K0-W	0.299 ± 0.023 a	0.723 ± 0.081 c	0.0325 ± 0.002 d	0.0109 ± 0.001 c	0.0151 ± 0.001 d
		K120-W	0.256 ± 0.027 d	0.671 ± 0.046 d	0.0338 ± 0.001 c	0.0105 ± 0.001 d	0.0162 ± 0.001 c
		K0-D	0.285 ± 0.028 b	0.774 ± 0.058 a	0.0416 ± 0.003 a	0.0127 ± 0.001 b	0.0178 ± 0.001 b
		K120-D	0.276 ± 0.027 c	0.759 ± 0.098 b	0.0361 ± 0.001 b	0.0129 ± 0.000 a	0.0185 ± 0.001 a
	2024	K0-W	0.277 ± 0.014 b	0.753 ± 0.047 a	0.0312 ± 0.001 c	0.0110 ± 0.000 c	0.0184 ± 0.001 c
		K120-W	0.264 ± 0.034 c	0.690 ± 0.075 c	0.0303 ± 0.001 d	0.0115 ± 0.001 b	0.0201 ± 0.002 a
		K0-D	0.287 ± 0.024 a	0.691 ± 0.058 c	0.0322 ± 0.001 b	0.0096 ± 0.000 d	0.0186 ± 0.001 c
		K120-D	0.259 ± 0.028 d	0.703 ± 0.090 b	0.0335 ± 0.002 a	0.0119 ± 0.001 a	0.0199 ± 0.001 b

Note: Data present means ± SE (*n* = 3). Different lowercase letters indicate significant differences (*p* < 0.05) among treatments within the same year and cultivar.

**Table 3:** Effect of K fertilization on grain quality traits in wheat.

Variety	Year	Treatment	Total Starch Content (%)	Protein Content (%)	Wet Gluten Content (%)
KX 3302	2023	K0-W	72.64 ± 1.36 b	14.18 ± 0.03 c	30.56 ± 0.07 b
		K120-W	74.66 ± 0.03 a	14.87 ± 0.03 a	31.98 ± 0.15 a
		K0-D	69.84 ± 0.37 c	14.70 ± 0.11 b	31.69 ± 0.22 a
		K120-D	74.18 ± 0.83 ab	14.94 ± 0.05 a	32.06 ± 0.31 a
	2024	K0-W	62.77 ± 0.04 c	15.95 ± 0.13 c	37.01 ± 2.79 c
		K120-W	67.43 ± 0.06 a	16.29 ± 0.10 b	38.35 ± 0.14 c
		K0-D	62.66 ± 0.07 c	16.15 ± 0.13 bc	38.81 ± 0.40 b
		K120-D	64.78 ± 0.01 b	16.63 ± 0.23 a	39.60 ± 0.26 a
KN 9204	2023	K0-W	64.69 ± 0.03 b	13.81 ± 0.14 c	30.65 ± 0.17 d
		K120-W	69.20 ± 0.02 c	14.71 ± 0.11 b	32.86 ± 0.08 b
		K0-D	61.97 ± 0.03 a	14.48 ± 0.10 a	31.63 ± 0.22 a
		K120-D	67.24 ± 0.01 a	15.73 ± 0.11 a	34.68 ± 0.27 c
	2024	K0-W	61.86 ± 0.35 c	14.15 ± 0.09 c	33.95 ± 0.14 c
		K120-W	75.83 ± 0.05 a	15.99 ± 0.14 a	37.67 ± 0.31 b
		K0-D	59.37 ± 0.53 d	14.92 ± 0.10 b	35.89 ± 0.39 c
		K120-D	75.44 ± 0.05 b	16.27 ± 0.29 a	38.74 ± 0.34 a

Note: Data present means ± SE (*n* = 3). Different lowercase letters indicate significant differences (*p* < 0.05) among treatments within the same year and cultivar.

## Data Availability

Data and material of this study are available from the corresponding author on reasonable request.

## Supplementary Materials

The following supporting information can be downloaded at: https://www.mdpi.com/article/10.3390/plants15040539/s1, Figure S1: Phenotypic responses and agronomic performance of wheat cultivars KX3302 and KN9204 at maturity under potassium fertilization and drought treatments. Figure S2: Correlation analysis between grain yield and multiple agronomic and quality traits in wheat cultivars KN9204 and KX3302. Table S1: Analysis of variance results for the effects of year, treatment, cultivar and their interactions on various parameters.
