# Supplementary material for: Application of Potassium Improves Yield and Quality Under Drought Stress by Regulating Nutrient Use Efficiency in Wheat
Source: Plants (Basel). 2026 Feb 9;15(4):539. doi: 10.3390/plants15040539 (PMC12944695; doi:10.3390/plants15040539)
Supplement: Supplementary file 1 [file plants-15-00539-s001.zip › Supplementary Figure-re.pdf]

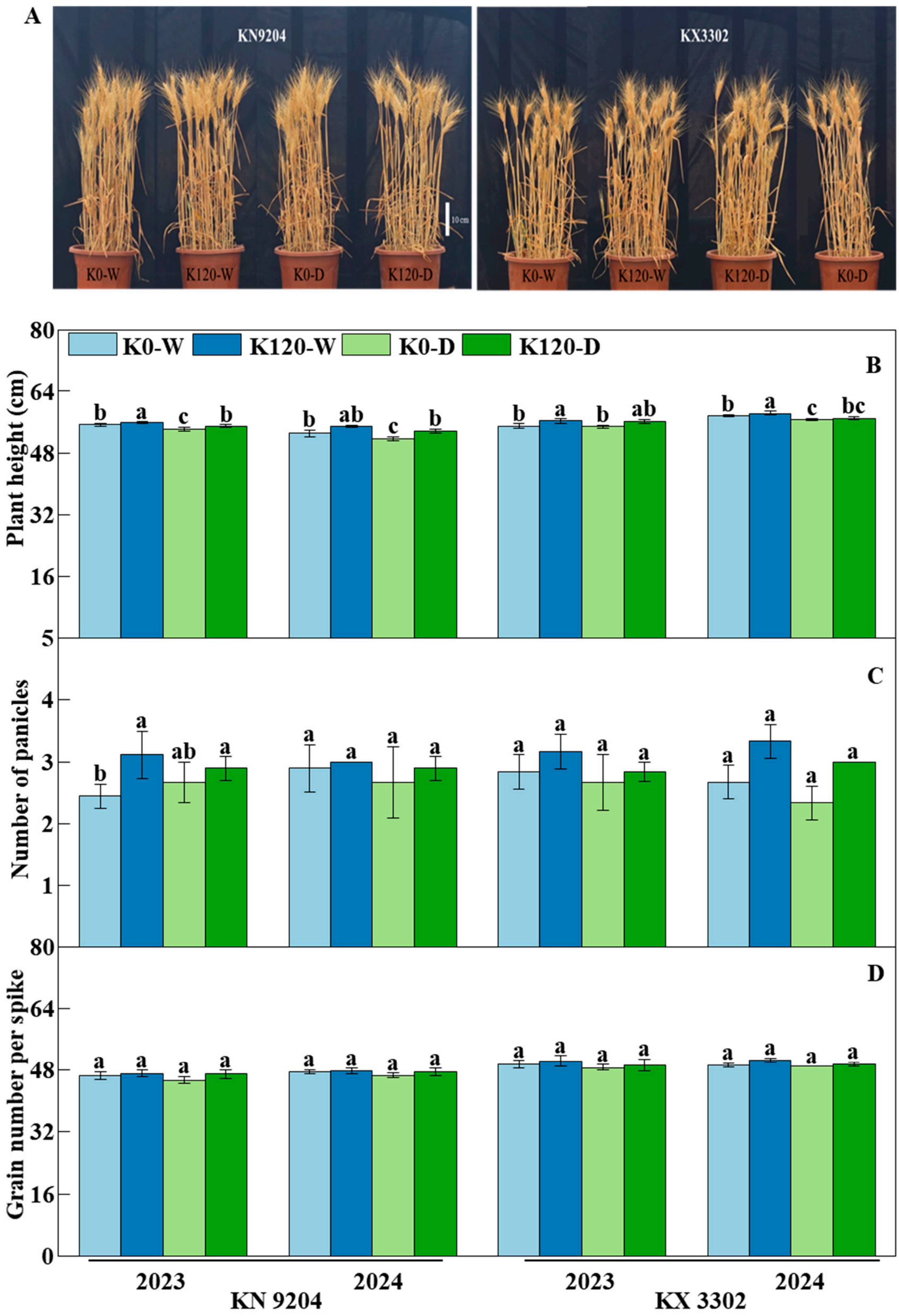

Supplementary Figure S1. Phenotypic responses and agronomic performance of wheat cultivars KX3302 and KN9204 at maturity under potassium and drought treatments. (A) Representative mature phenotypes of KX3302 and KN9204 under well-watered (W) and post-flowering drought stress (D) conditions with or without potassium application (K120 or K0). Bar = 10 cm. (B) Plant height of both cultivars under four treatment combinations (K0-W, K120-W, K0-D, and K120-D)

in 2023 and 2024. (C) Spike number per plant for the same treatments and years. (D) Grain number per spike under the four treatments across both years. Data represent means ± SE (n = 3), and different letters indicate significant differences at $P < 0.05$.

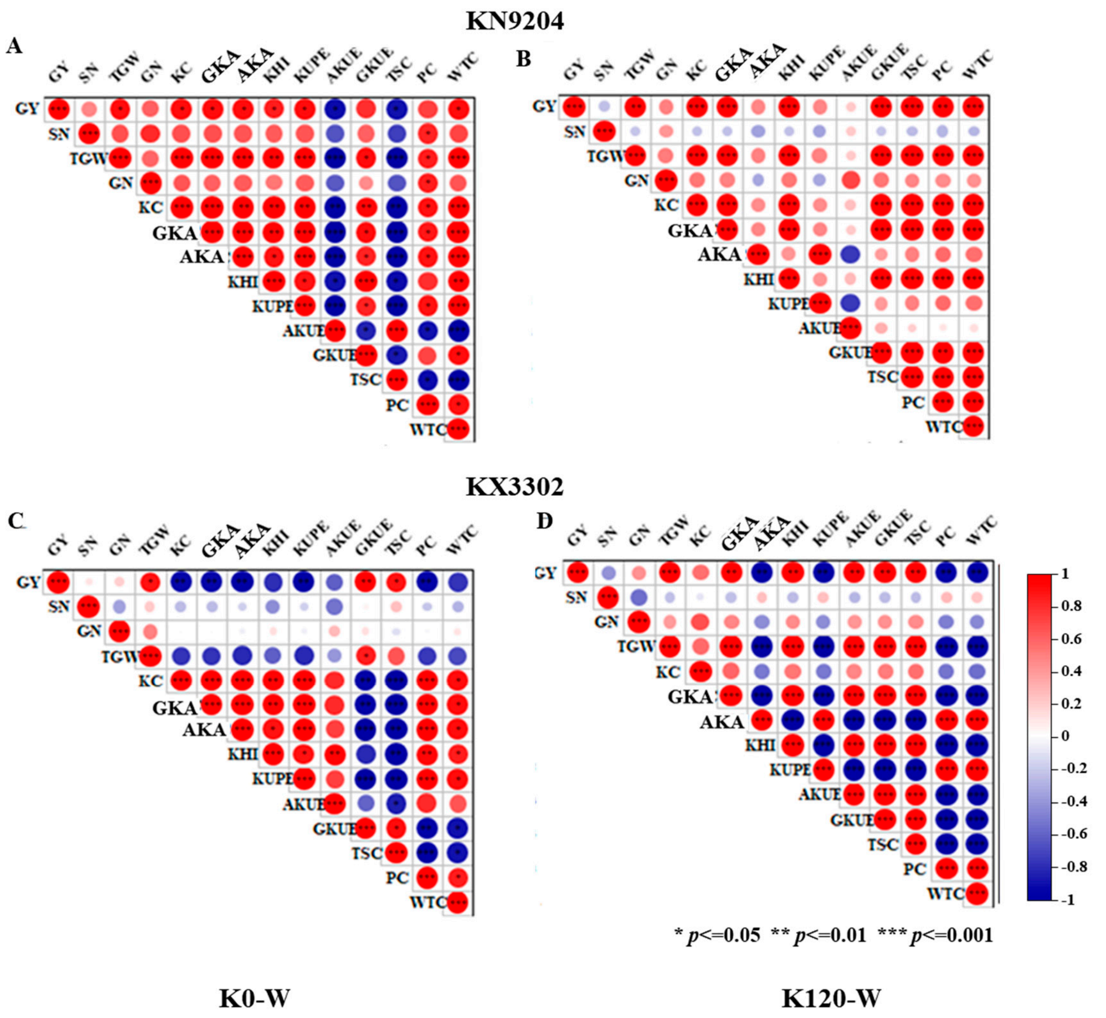

Supplementary Figure S2. Correlation analysis between grain yield and multiple agronomic and characteristic traits in wheat cultivars KN9204 and KX3302. Panels (A–B) show results for KN9204 under K0–W, and K120–W, respectively; panels (C–D) present corresponding data for KX3302 under the same treatments. Significant correlations ($P < 0.05$) are color-coded (red for positive, blue for negative), with circle size proportional to the absolute value of the correlation coefficient (r).
